# Systemic Salicylate Toxicity From Topical Emollient Use in an Infant With Suspected Ichthyosis

**DOI:** 10.7759/cureus.110778

**Published:** 2026-06-13

**Authors:** Kevin Pullukat, Tarek Husien

**Affiliations:** 1 Department of Pediatrics, Detroit Medical Center (DMC) Children's Hospital of Michigan, Detroit, USA; 2 School of Medicine, Central Michigan University College of Medicine, Mount Pleasant, USA

**Keywords:** atopic dermatitis, icthyosis, infant, pediatric skin conditions, salicylate toxicity, sensitive skin, skin barrier, toxicology

## Abstract

We report a case of a six-month-old female infant weighing 4.56 kg who presented with altered mental status, severe metabolic acidosis, and multiorgan dysfunction following chronic use of an over-the-counter topical cream containing salicylic acid. The patient had a longstanding pruritic rash treated at home with multiple daily applications of the same topical salicylic acid-containing cream. She developed symptoms consistent with salicylate toxicity in the absence of known ingestion, confirmed by an elevated serum salicylate concentration. Intensive care was required to manage profound metabolic derangements, hepatic dysfunction, coagulopathy, thrombocytopenia, and hypoalbuminemia with third-spacing. Management included intravenous fluids, calcium gluconate, sodium bicarbonate therapy with urine alkalinization, potassium optimization, and supportive care. Dermatology evaluation raised concern for ichthyosis versus severe atopic dermatitis, and genetic testing was nondiagnostic except for a variant of uncertain significance in SLC27A4. The patient improved without dialysis and was discharged with dermatologic care instructions and close subspecialty follow-up. This case highlights the risk of systemic toxicity from topical salicylates in infants, particularly in the presence of impaired skin barrier function, and underscores the importance of cautious use of over-the-counter topical products in vulnerable pediatric populations.

## Introduction

Salicylic acid is a keratolytic agent commonly found in a variety of over-the-counter dermatologic products, including acne treatments, wart removers, dandruff shampoos, and moisturizers. Salicylic acid concentrations vary by product type; for over-the-counter acne products in the United States, concentrations up to 2% are permitted [1]. Although these products are generally considered safe when used as directed in appropriate populations, topical salicylic acid products are generally not recommended for routine use in young infants because of the potential for increased systemic absorption and toxicity [2]. Salicylate toxicity can occur with inappropriate use, excessive application, or exposure through compromised skin barriers [3].

Infants are especially vulnerable to percutaneous toxicity because of their increased body surface area-to-weight ratio, thinner stratum corneum, and immature renal and hepatic clearance mechanisms [3,4]. This risk may be further increased by chronic inflammation, diffuse skin involvement, undiagnosed disorders of cornification, or occlusive practices such as swaddling after topical application. Prior pediatric reports of topical salicylate toxicity have most often involved higher-concentration compounded preparations or children with underlying skin barrier disease [5-8].

We present a case of systemic salicylate toxicity in a six-month-old infant following prolonged use of an over-the-counter 2% salicylic acid cream. This case emphasizes that clinically significant toxicity may occur even without known ingestion and highlights the importance of asking specifically about topical products in infants with unexplained metabolic acidosis or systemic illness.

## Case presentation

A six-month-old unvaccinated female infant weighing 4.56 kg with no significant past medical history presented to the emergency department for evaluation of poor feeding, emesis, and progressive lethargy. The patient had a persistent, pruritic, dry rash since three months of age, initially appearing on the face and later spreading caudally. In the absence of routine pediatric care, caregivers had been applying an over-the-counter product containing 2% salicylic acid multiple times daily for several months, as well as calendula oil/vitamin E. Caregivers also reported use of oral teething drops containing herbal ingredients, including clove and spearmint oils. The most recent application of the cream occurred on the morning of presentation.

Over the several days preceding presentation, the patient developed decreased oral intake and progressive lethargy. On the day of presentation, she had increased irritability and vomiting following the routine application of topical products. In the emergency department, she was afebrile but ill-appearing, listless, and clinically dehydrated, with dry mucous membranes and sunken eyes. She was tachypneic, tachycardic to 204 beats/min, and hypotensive to 69/25 mmHg. Physical examination was also notable for a diffuse erythematous dry scaly rash without vesicles or pustules. Initial venous blood gas demonstrated pH 6.98, bicarbonate 5.7 mmol/L, and pCO₂ 22 mmHg, consistent with severe metabolic acidosis. Lactate was elevated at 12.15 mmol/L. Given her altered mental status and profound acidosis, the initial evaluation included infectious, metabolic, and toxicologic testing. Head computed tomography was unremarkable, viral testing was negative, and urine drug screening was negative. Toxicologic testing was notable for an elevated serum salicylate concentration of 31 mg/dL. This result prompted further review of topical and over-the-counter exposures, including use of the salicylic acid-containing cream on the morning of presentation. She had hyperkalemia with potassium 6.2 mmol/L, and peaked T waves were seen on cardiac monitoring. Key laboratory abnormalities are summarized in Table 1.

**Table 1:** Key Laboratory Abnormalities During Hospitalization Values reflect initial presentation unless otherwise specified.

Laboratory Test	Patient Value	Reference Range
Venous pH	6.98	7.35–7.45
Bicarbonate (mmol/L)	5.7	22–28
pCO₂ (mmHg)	22	35–45
Lactate (mmol/L)	12.15	0.5–2.2
Salicylate concentration (mg/dL)	31	<6
Potassium (mmol/L)	6.2	3.5–5.1
Albumin (g/dL)	2.6	2.8–4.8
Platelet count, nadir (×10³/µL)	46	130–450
PT (seconds)	86.8	9.4–11.7
INR	>9.36	0.86–1.11

Initial management included intravenous fluid resuscitation, calcium gluconate for cardioprotection, and intravenous sodium bicarbonate to promote urinary alkalinization. She was also started on high-flow nasal cannula and transferred to the pediatric intensive care unit for further management. Toxicology recommended continuation of bicarbonate therapy with careful monitoring of serum and urinary pH, maintaining potassium levels above 4 mmol/L to support urine alkalinization, and avoiding endotracheal intubation when possible because loss of compensatory hyperventilation can worsen acidemia.

During the PICU course, a Foley catheter was placed for strict intake/output monitoring and serial urine pH assessment during alkalinization therapy. Early in her PICU course, urine output decreased to 23 mL over 24 hours, approximately 0.2 mL/kg/hr based on her admission weight. She developed worsening coagulopathy, hypoalbuminemia with third-spacing, and transaminitis, with peak AST 95 U/L (reference range: 13-39 U/L) and ALT 134 U/L (reference range: 7-52 U/L). Coagulation studies were markedly abnormal, with PT 86.8 seconds, INR >9.36, and fibrinogen later decreasing to 80 mg/dL. The patient also developed thrombocytopenia, with platelet count decreasing to a nadir of 46 ×10³/µL during hospitalization. Despite marked coagulopathy and thrombocytopenia, no bleeding was reported. She was treated with vitamin K 2.5 mg daily for three days and cryoprecipitate at 10 mL/kg. Hypoalbuminemia with third-spacing was treated with four doses of 25% albumin at 1 g/kg per dose followed by furosemide at 1 mg/kg.

Hematology and toxicology felt the coagulopathy was likely multifactorial, with suspected contributions from salicylate toxicity and possible contribution from herbal exposures, including clove oil/eugenol and vitamin E. Workup was negative for hemolysis, with a normal direct antiglobulin test and only mild elevation in lactate dehydrogenase. As part of the hematology evaluation for thrombocytopenia, echocardiogram and abdominal/pelvic duplex ultrasound were obtained to assess for thrombosis and were unremarkable.

Dermatology was consulted during admission and raised concern for ichthyosis versus severe atopic dermatitis. They recommended discontinuation of salicylic acid-containing products and treatment with topical corticosteroids, bland emollients, and nightly wet wraps. Genetic testing was sent to evaluate for inherited ichthyosis and later revealed a variant of uncertain significance in SLC27A4. This variant was felt unlikely to explain the phenotype because SLC27A4-associated ichthyosis prematurity syndrome is typically autosomal recessive and characterized by prematurity, thick desquamating epidermis at birth, and neonatal respiratory complications, none of which were present in this patient [9].

Within 24 hours of admission, her mental status had improved, with examination showing that she was awake and alert without focal neurologic deficits. Her pH normalized with bicarbonate therapy, and her salicylate concentration declined from 31 mg/dL on admission to 18 mg/dL within 24 hours and 7.4 mg/dL within 48 hours. She was later transferred to the general pediatric ward after stabilization and transitioned to full oral feeds. Prior to discharge, her platelet count had increased to 124 ×10³/µL and albumin had improved to 2.9 g/dL. She was discharged with dermatologic care instructions, ergocalciferol for vitamin D deficiency, and close follow-up with dermatology, hematology/oncology, genetics, and primary care. On outpatient follow-up, her platelet count, PT, aPTT, and fibrinogen had normalized to 251 ×10³/µL, 10.5 seconds, 25.2 seconds, and 219 mg/dL, respectively, supporting a transient acquired coagulopathy related to the acute toxicity. Subsequent outpatient genetic evaluation remained nondiagnostic. 

## Discussion

Salicylate toxicity in children is classically associated with ingestion. However, rare but serious reports describe systemic toxicity from percutaneous absorption, particularly in children with predisposing dermatologic conditions or exposure to compounded or higher-concentration topical preparations. Prior reports by Germann et al., Chiaretti et al., Fil et al., and Oualha et al. described topical salicylate toxicity in pediatric patients involving salicylic acid preparations ranging from 10% to 23%, often in the setting of underlying disorders of skin barrier function, such as ichthyosis or severe dermatitis [5-8].

This case adds to the literature by describing severe systemic salicylate toxicity in an infant after chronic exposure to a commercially available over-the-counter cream containing 2% salicylic acid. To our knowledge, few reports describe life-threatening toxicity associated with chronic use of commercially available over-the-counter 2% salicylic acid preparations in infancy. Notably, this occurred in the absence of known ingestion or a prior diagnosis of ichthyosis, suggesting that undiagnosed or subclinical skin barrier disorders may represent an underrecognized risk factor for systemic toxicity even with lower-concentration formulations.

Several factors likely contributed to toxicity in this patient. Infants have an increased body surface area-to-weight ratio, and this patient had repeated topical exposure over diffusely inflamed, dry, scaly skin, although a specific body surface area percentage was not documented. Occlusion or swaddling after topical application may further increase absorption by raising local skin temperature and prolonging contact with the applied product. Impaired epidermal barrier function from suspected ichthyosis or severe atopic dermatitis likely increased systemic absorption. Chronic and liberal application may have further increased cumulative exposure.

This case also illustrates the clinical severity of salicylate toxicity, which manifested as profound metabolic acidosis, hyperkalemia with electrocardiographic changes, hepatic dysfunction, coagulopathy, thrombocytopenia, hypoalbuminemia, and third-spacing. The patient’s serum salicylate concentration of 31 mg/dL was elevated but lower than levels often reported in severe acute ingestions, reinforcing that clinical severity may not correlate perfectly with a single measured salicylate level, particularly in infants and in cases of delayed, chronic, or ongoing percutaneous absorption and immature renal or hepatic elimination [3].

Toxicology guidance was central to management. Sodium bicarbonate therapy promotes serum and urine alkalinization, which reduces tissue penetration of salicylate and enhances renal elimination [3]. Potassium correction is also essential because hypokalemia can impair urine alkalinization. Airway management requires caution because intubation may remove the patient’s compensatory hyperventilation and precipitate worsening acidemia if minute ventilation is not carefully maintained [3].

This case has several limitations. The exact quantity of the cream applied was not documented, which limits assessment of a dose-response relationship between topical salicylic acid exposure and systemic toxicity. In addition, exposure to other topical and oral products, including clove oil/eugenol and vitamin E, may have contributed to hepatic dysfunction or coagulopathy, making it difficult to attribute all systemic findings solely to salicylate toxicity. Future reports of topical salicylate toxicity should include detailed documentation of quantity applied, frequency and duration of use, and estimated body surface area exposed, to better characterize risk in infants with impaired skin barriers.

From a systems-level perspective, this patient’s lack of an established medical home and unmonitored use of topical products highlight the preventive role of routine pediatric care, caregiver education, and clinician vigilance regarding over-the-counter treatments in infants. Similar branding among non-medicated moisturizers and salicylic acid-containing formulations may also contribute to caregiver confusion, underscoring the importance of reviewing exact product names and active ingredients during medication and exposure histories. Even products that are generally safe when used as directed may carry significant risk in vulnerable populations, particularly when applied repeatedly over broad areas to compromised skin.

## Conclusions

Topical salicylate products can cause life-threatening systemic toxicity in infants, especially when applied chronically to inflamed or disrupted skin. Salicylate-containing over-the-counter products should generally be avoided in infants, particularly those with compromised skin barriers.

Clinicians should consider percutaneous salicylate toxicity in infants presenting with unexplained metabolic acidosis, altered mental status, or multisystem illness and should ask specifically about over-the-counter topical products. Early toxicology consultation, bicarbonate therapy, potassium optimization, and careful airway planning are critical. Early dermatologic evaluation and caregiver education are also essential to prevent inappropriate use of keratolytic products in infants. Clearer caregiver-facing labeling regarding pediatric age restrictions may further reduce the risk of inadvertent exposure.

## References

[1] CFR 333.310 (2025). Acne active ingredients. https://www.ecfr.gov/current/title-21/chapter-I/subchapter-D/part-333/subpart-D/section-333.310.

[2] (2026). U.S. Food and Drug Administration: Beta hydroxy acids. https://www.fda.gov/cosmetics/cosmetic-ingredients/beta-hydroxy-acids.

[3] Runde TJ, Nappe TM (2026). Salicylates toxicity. StatPearls [Internet].

[4] Fairley JA, Rasmussen JE (1983). Comparison of stratum corneum thickness in children and adults. J Am Acad Dermatol.

[5] Germann R, Schindera I, Kuch M, Seitz U, Altmeyer S, Schindera F (1996). Life threatening salicylate poisoning caused by percutaneous absorption in severe ichthyosis vulgaris (Article in German). Hautarzt.

[6] Chiaretti A, Schembri Wismayer D, Tortorolo L, Piastra M, Polidori G (1997). Salicylate intoxication using a skin ointment. Acta Paediatr.

[7] Fil E, Dilek S, Umur Ö, Dag H (2024). An infant developed intoxication following topical salicylate use: a case report. Pediatr Acad Case Rep.

[8] Oualha M, Dupic L, Bastian C, Bergounioux J, Bodemer C, Lesage F (2012). Local salicylate transcutaneous absorption: an unrecognized risk of severe intoxication: a case report (Article in French). Arch Pediatr.

[9] Al-Khenaizan S, Al-Sannaa N, Al-Turaiki H (2021). Ichthyosis prematurity syndrome: a rare form but easily recognized. Cureus.

